# Comorbid mental disorders during long‐term course in a nationwide cohort of patients with anorexia nervosa

**DOI:** 10.1002/eat.23570

**Published:** 2021-06-18

**Authors:** Hans‐Christoph Steinhausen, Martin Dalgaard Villumsen, Kirsten Hørder, Laura Al‐Dakhiel Winkler, Niels Bilenberg, René Klinkby Støving

**Affiliations:** ^1^ Department of Child and Adolescent Mental Health Odense, Mental Health Services in the Region of Southern Denmark University of Southern Denmark Odense Denmark; ^2^ Child and Adolescent Mental Health Centre Capital Region Psychiatry Copenhagen Denmark; ^3^ Department of Child and Adolescent Psychiatry Psychiatric University Hospital of Zurich Switzerland; ^4^ Clinical Psychology and Epidemiology, Institute of Psychology University of Basel Switzerland; ^5^ Department of Epidemiology, Biostatistics, and Biodemography, Institute of Public Health University of Southern Denmark Odense Denmark; ^6^ Center for Eating Disorders Odense University Hospital Odense Denmark; ^7^ OPEN – Open Patient Data Explorative Network Odense University Hospital Denmark; ^8^ Psychiatric Services in the Region of Southern Denmark Odense Denmark; ^9^ Endocrine Research Unit Odense University Hospital Denmark

**Keywords:** anorexia nervosa, comorbidity, epidemiology, matched cohort design, register data

## Abstract

**Objective:**

Comorbid mental disorders in anorexia nervosa during long‐term course require detailed studies.

**Method:**

This matched cohort study was based on nationwide Danish register data of all patients born 1961–2008 with a first‐time ICD‐10 diagnosis of anorexia nervosa (AN) between 1994 and 2018 at age 8–32 and matched controls taken from all individuals without an eating disorder (ED). For nine categories of non‐eating mental disorders, time from date of first AN‐diagnosis (inclusion date) to time of first diagnosis, accounting for censoring, was studied by use of time‐stratified Cox models.

**Results:**

A total of 9,985 patients with AN (93.5% females) and 49,351 matched controls were followed for a median (IQR) of 9.0 (4.4–15.7) years. For patients, there was about 25% and 55% risk of receiving any non‐ED disorder during the first 2 years and two decades after inclusion, respectively. A hazard ratio (HR) of seven for any non‐ED was found for the first 12 months after inclusion, a ratio that reduced to two at five or more years after inclusion. During the first years, large HRs ranging in 6–9 were found for affective, autism spectrum, personality, and obsessive–compulsive disorders with the latter displaying the highest continuous increased risk. The HR at 12 months after inclusion was highest for any non‐ED disorder and affective disorders in patients aged 8–13 at diagnosis.

**Discussion:**

Comorbid mental disorders in AN are most frequently diagnosed in the first years after diagnosis of AN and on longer terms imply a double immediate risk.

## 1 INTRODUCTION

Anorexia nervosa (AN) commonly cooccurs with other mental disorders (Demmler, Brophy, Marchant, John, & Tan, 2020; Hudson, Hiripi, Pope, & Kessler, 2007; Keski‐Rahkonen & Mustelin, 2016; Tsai et al., 2018; Udo & Grilo, 2019; Ulfvebrand, Birgegard, Norring, Hogdahl, & von Hausswolff‐Juhlin, 2015). These most commonly include anxiety disorders (Meier et al., 2015), depression (Carrot et al., 2017), obsessive–compulsive disorders (OCD) (Meier et al., 2015; Yilmaz et al., 2020), substance use disorders (Fouladi et al., 2015; Root et al., 2010), personality disorders (Martinussen et al., 2017; von Lojewski, Fisher, & Abraham, 2013), autism spectrum disorders (Koch et al., 2015; Wentz, Gillberg, Anckarsater, Gillberg, & Rastam, 2009; Westwood & Tchanturia, 2017), and attention‐deficit‐hyperactivity disorder (Nazar et al., 2016). In addition, mortality is markedly increased in AN with the presence of mental health comorbidity (Himmerich et al., 2019; Kask et al., 2016).

A systematic review of all long‐term follow‐up studies of the clinical course of AN performed in the 20th century reported a variety of comorbid disorders including anxiety disorders and phobias (25.5%), affective disorders (24.1%), substance use disorders (14.4%), obsessive–compulsive disorders (12.2%), and various personality disorders (16.6–31.4%) (Steinhausen, 2002). More recent follow‐up studies have revealed that comorbid depression at the start of treatment is a negative predictor of outcome for the restrictive type of AN (Eskild‐Jensen, Stoving, Flindt, & Sjogren, 2020; Franko et al., 2018), and that OCD is both a frequent life‐time and follow‐up diagnosis (Mandelli, Draghetti, Albert, De Ronchi, & Atti, 2020). Both OCD (Mandelli et al., 2020) and autistic traits are also associated with a less favorable outcome (Wentz et al., 2009). At the 5–10‐year follow‐up, 28% of patients with a childhood onset of AN had a current comorbidity and 64% had a past comorbid diagnosis (Herpertz‐Dahlmann et al., 2018).

Age of onset has been shown to have an effect on outcome. In particular, onset of AN during adolescence was associated with a more benign outcome in a sizeable number of outcome studies (Steinhausen, 2002; Steinhausen, Boyadjieva, Griogoroiu‐Serbanescu, & Neumarker, 2003). None of these studies considered the association between AN and comorbid mental disorders concurrent or subsequent to the AN diagnosis as outcomes. Further, previous research has not accounted for the risk in controls, implying that the reported results may to some degree reflect common challenges in adolescent life.

The present report is part of a multi‐domain study of the long‐term course of a nationwide Danish cohort of patients with AN compared to matched controls. In the entire set of analyses, a specific focus is on the effect of age at diagnosis as the best proxy of age at onset of AN. The aim of this study was to investigate whether concurrent and subsequent mental disorders during long‐term follow‐up are more frequent and differ across time in AN patients compared to controls, and whether age at diagnosis of AN modifies the association.

## 2 METHOD

### 2.1 Study samples

The study was based on the complete nationwide cohort of all individuals born 1961–2008 who were diagnosed with an eating disorder (ED) in 1994–2018 and at the time of the first diagnosis were at age 8–32 years. EDs were defined by the International Classification of Diseases and Related Health Problems, 10th edition (WHO, 1992), code F50 in the National Patient Register (NPR). Patients with AN (F50.0, F50.1) were identified in the registers as individuals with at least one AN diagnosis at the first day of registration with a non‐emergency incident ED diagnosis, regardless of whether the patient simultaneously had a diagnosis of bulimia nervosa. The age range of the sample was guided by the idea that the validity of the diagnosis of AN is uncertain outside this age range. Based on studies analyzing childhood AN (see Herpertz‐Dahlmann et al., 2018), we decided to truncate the empirical distribution at the lower age of 8 years at first diagnosis. The upper age at first diagnosis limitation was guided by the following considerations. First, it is questionable whether late onset exists or is due to late diagnosis (Scholtz, Hill, & Lacey, 2010). Secondly, in a previous study on life‐time incidence rates of first‐time diagnosed AN up to age 65 based on Danish registry data, we found a sharp decline of incidences starting with the age‐group 30–39 (Scholtz et al., 2010; Steinhausen & Jensen, 2015).

To avoid prevalent cases from being classified as incident cases, all patients with access to services before 1994 were excluded by study design. This procedure, which was used separately for each disorder, implies an exclusion of all patients and controls with the ICD‐8 classification. In Denmark, the ICD‐8 classification was used from 1969 to 1993, the ICD‐9 was never adopted, and the ICD‐10 was used from 1994.

Using a matched design with a 1:5 ratio, controls alive at the time of inclusion of matched patients were identified via the Danish Civil Registration System (DCRS), which covers the total general population. The date of the first AN‐diagnosis defines the inclusion date for both the patients and the controls. The matching was done on sex, age, and area of residence at the time of the first eating disorder diagnosis in the patients. By design, controls did not have any ED diagnosis prior to or at any time after inclusion. All participants were alive, and resided in Denmark at the initiation of follow‐up.

**FIGURE 1:**
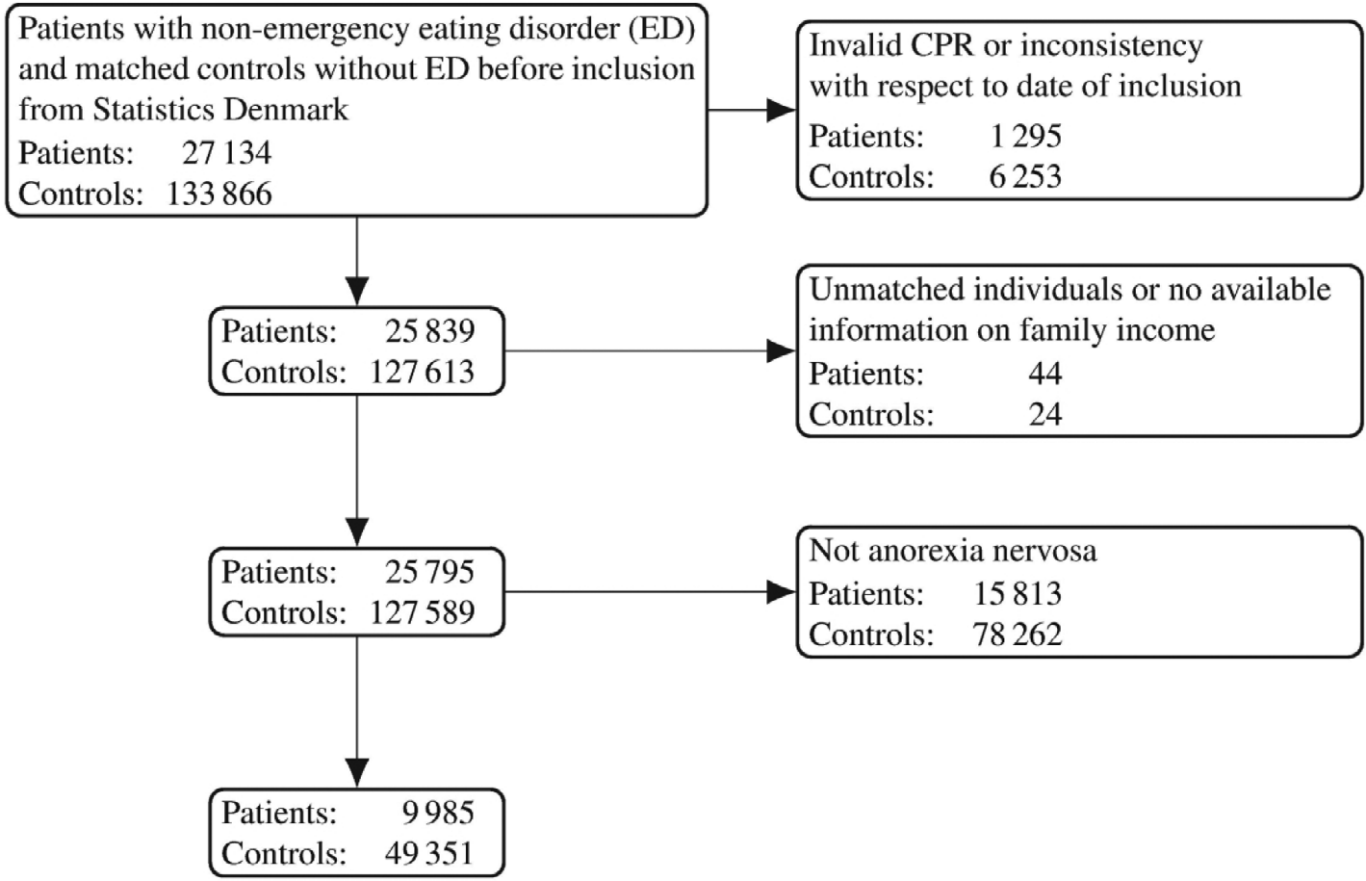
Flowchart for identification of the study population

Figure 1 presents a flowchart of the sample populations. Starting from 27,134 individuals with an eating disorder and 133,866 controls, the final sample amounted to 9,985 patients with anorexia nervosa (F 50.0 anorexia nervosa and F 50.1 atypical anorexia nervosa) and to 49,351 controls. The sample was divided into three subgroups by age in years at first diagnosis of anorexia nervosa, covering 8–13, 14–17, and 18–32 at the time of inclusion. The three subgroups were defined according to common developmental distinctions between childhood including preadolescence (age 8–13), adolescence (age 14–17), and adulthood (age 18 and older).

### 2.2 Procedure

The study was based on the comprehensive Danish system of registries, which includes mandatory recording of patient visits to the public health service. The system covers various domains of data collection including extensive health information on the total national population since 1968 (Pedersen, 2011). In the present study, based on the personal identification number (CPR) assigned to all Danish citizens and residents, information was collected from the DCRS on date of birth, sex, postcode of residence at the time of the first diagnosis in the NPR. Furthermore, data on mental disorders including first‐time AN diagnosis, time and type of admission to psychiatric facilities, and information regarding parental mental health were collected from the Danish Psychiatric Central Research Register (DPCRR) (Mors, Perto, & Mortensen, 2011). Additional information on family income as an indicator of socioeconomic status was collected from the Income Statistics Register. Data were provided by Statistics Denmark in an anonymized fashion and approval of the study was given by the Danish Data Protection Agency (file no. 15/280490). According to Danish law, ethical approval is not required for registry‐based studies. Access to the data requires application to the Danish authorities.

### 2.3 Diagnoses

With a focus on concurrent and subsequent mental disorders in AN‐patients and matched controls during long‐term follow‐up, the following nine major categories of mental disorders in the International Classification of Diseases (WHO, 1992) were considered in the analyses: any non‐ED (F00–F49 + F51–F99), substance use disorders (F10–F19), schizophrenia or psychosis (F20–F29), affective disorders (F38–F39), phobia or anxiety disorders (F40, F41), obsessive–compulsive disorders (F42), adjustment disorders (F43), personality disorders (F60–F69), and autism spectrum disorders (F84.0 + F84.1 + F84.5). Frequencies of non‐emergency incident disorders were obtained both for the two‐year period prior to inclusion and during follow‐up. All diagnoses were made by the attending physicians.

### 2.4 Statistical analyses

Using Cox proportional hazards models with cluster robust standard errors, time from first AN‐diagnosis to first diagnosis, accounting for censoring, was investigated for each of the nine mental disorders on the matched cohort data. Separate effects were estimated on periods for which time‐invariance was assessed with Aalen's linear hazard models (Lee & Weissfeld, 1998). The Cox models were adjusted for previous mental diagnosis during the 2 years prior to inclusion, sex, study period at time of inclusion (1994–1999, 2000–2004, 2005–2009, 2010–2014, 2015–2018), family income (in tertiles), and parental previous mental disorder at the time of inclusion. The assumptions of proportional hazards were verified via Schoenfeld residuals tests. Tests for interaction between age group and anorexia nervosa (patients vs. controls) for each mental disorder were conducted. The method proposed by Holm (1979) was used to correct for multiple testing. The risk of each of the nine mental disorders over time in patients and controls were compared using cumulative incidences, with death as competing risk, for each age‐subgroups as well as the combined sample. To guarantee anonymity of individuals, cumulative incidences are graphed after applying a multiplicative distortion of a Gaussian distributed random error with mean 1 and *SD* 0.05. All analyses were performed with Stata 16.1 (Statacorp, College Station, TX) on servers at Statistics Denmark.

## 3 RESULTS

The baseline characteristics of each group are shown in Table 1. Patients with anorexia nervosa were predominantly female (93.5%). The largest age group (45%) was 18–32 years old, while 38% were 14–17 and 17% were 8–13 years old. Median (IQR) follow‐up time in years for the two samples was 9.0 (4.4–15.7). At the time of study inclusion, the AN sample consisted of 1,349 (13.5%) inpatients, 8, 595 (86.1%) outpatients and a negligible number of 41 patients in a day‐clinic (0.4%). As controls were matched on age listed in years, the age at inclusion for controls exceeds the age range 8–32 (range 7.6–33.9). A higher proportion of patients with AN than controls (12.7% vs. 3.6%) had a mental diagnosis in the 2 years prior to inclusion, but frequencies of the various other mental disorders in the two samples prior to inclusion were rather low. Among patients and controls, 0.7% respectively 0.2% were lost because of death.

**TABLE 1:** Sample characteristics

	Patients	Controls
*Total*	9,985	49,351
*Sex*, no. (%)		
Female	9,331 (93.5)	46,103 (93.4)
Male	654 (6.5)	3,248 (6.6)
*Age in years at inclusion*, median (IQR)	17.3 (14.7–21.3)	17.3 (14.7–21.3)
*Age groups in years*		
8–13	1724 (17.3)	8,519 (17.3)
14–17	3,809 (38.1)	18,848 (38.2)
18–32	4,452 (44.6)	21,984 (44.5)
*Previous other mental disorders*, no. (%)		
Any non‐ED disorder	1,273 (12.7)	1759 (3.6)
Substance use disorders	159 (1.6)	334 (0.7)
Schizophrenia or psychosis	121 (1.2)	145 (0.3)
Affective disorders, no. (%)	391 (3.9)	414 (0.8)
Phobia and anxiety disorders	159 (1.6)	243 (0.5)
Obsessive–compulsive disorders	101 (1.0)	91 (0.2)
Adjustment disorders	409 (4.1)	546 (1.1)
Personality disorders	234 (2.3)	244 (0.5)
Autism spectrum disorders	58 (0.6)	87 (0.2)
*Family income group* (tertiles)		
Lower tertile	3,477 (34.8)	16,301 (33.0)
Middle tertile	3,011 (30.2)	16,768 (34.0)
Higher tertile	3,497 (35.0)	16,282 (33.0)
*Previous mental disorders of parents*, no. (%)		
None	7,827 (78.4)	40,150 (81.4)
1+ diagnoses in single parent	1890 (18.9)	8,007 (16.2)
1+ diagnoses in both parent	268 (2.7)	1,194 (2.4)
*Period of inclusion*, no. (%)		
1994–1999	1,441 (14.4)	7,075 (14.3)
2000–2004	1,595 (16.0)	7,835 (15.9)
2005–2009	1971 (19.7)	9,726 (19.7)
2010–2014	2,725 (27.3)	13,537 (27.4)
2015–2018	2,253 (22.6)	11,178 (22.6)

**FIGURE 2:**
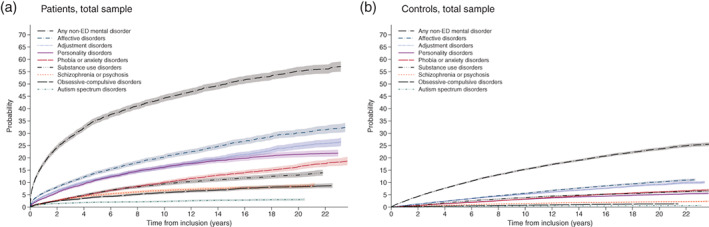
Cumulative incidences for other mental disorders of patients with AN and Controls in the two samples

**FIGURE 3:**
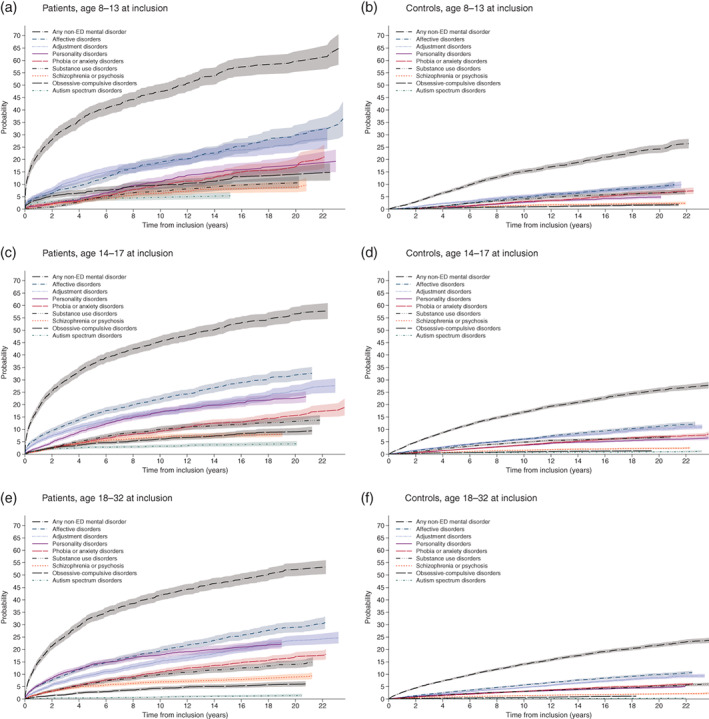
Cumulative incidences for other mental disorders of patients with AN and Controls stratified for age

For documentation, regardless of censored individuals, the frequencies of mental disorders other than AN for the entire observation period for the total samples and the various age groups are listed in Table S1. Cumulative incidence curves for each of the nine mental disorders for patients and controls are collected with Figure 2 for the total sample and with Figure 3 for the three age groups 8–13, 14–17, and 18–32. Table 2 lists cumulative incidences at selected time points for non‐ED disorders in the total and age stratified samples of patients and controls. More than double cumulative incidence was observed at any time during follow‐up for patients when compared to controls in each stratum. For example, at 4, 10, and 22 years the cumulative incidence (95% CI) for the total sample of patients was 32.1% (31.0%–33.3%), 44.3% (42.9%–45.6%), and 56.1% (54.2%–57.9%), while for controls it was 7.70% (7.46%–7.95%), 15.4 (15.0%–15.7%), and 25.0% (24.3%–25.6%).

**TABLE 2:** Cumulative incidences for non‐eating disorders in the total sample and in the three age groups

	Cumulative incidence (95% CI), (%)		Cumulative incidence (95% CI), (%)		Cumulative incidence (95% CI), (%)		Cumulative incidence (95% CI), (%)	
	All		8–13		14–17		18–32	
Years after inclusion	Patients	Controls	Patients	Controls	Patients	Controls	Patients	Controls
0.5	13.0 (12.2–13.8)	1.38 (1.28–1.48)	17.7 (15.7–19.8)	0.774 (0.604–0.980)	13.8 (12.6–15.2)	1.38 (1.22–1.55)	10.2 (9.11–11.3)	1.60 (1.44–1.78)
1	17.7 (16.8–18.6)	2.39 (2.25–2.53)	21.9 (19.7–24.2)	1.40 (1.17–1.67)	18.7 (17.2–20.2)	2.51 (2.29–2.75)	15.0 (13.7–16.3)	2.66 (2.45–2.88)
2	24.2 (23.2–25.2)	4.33 (4.15–4.52)	28.1 (25.6–30.6)	2.93 (2.57–3.32)	25.4 (23.8–27.1)	4.82 (4.53–5.16)	21.4 (19.9–22.9)	4.43 (4.16–4.72)
4	32.1 (31.0–33.3)	7.70 (7.46–7.95)	35.8 (33.1–38.5)	6.51 (5.96–7.09)	33.3 (31.5–35.2)	8.70 (8.28–9.13)	29.5 (27.8–31.2)	7.31 (6.95–7.67)
6	37.6 (36.4–38.8)	10.6 (10.3–10.9)	40.7 (37.8–43.5)	9.86 (9.16–10.6)	39.0 (36.7–40.9)	11.9 (11.4–12.5)	35.1 (33.2–36.9)	9.78 (9.35–10.2)
8	41.1 (39.8–42.4)	13.2 (12.8–13.5)	44.1 (41.2–47.1)	12.8 (12.0–13.7)	42.4 (40.3–44.4)	14.7 (14.1–15.3)	38.6 (36.6–40.5)	12.0 (11.5–12.5)
10	44.3 (42.9–45.6)	15.4 (15.0–15.7)	47.3 (44.2–50.4)	15.2 (14.3–16.2)	45.3 (43.2–47.4)	17.0 (16.4–17.6)	41.9 (39.8–43.9)	14.0 (13.5–14.6)
12	46.8 (45.4–48.2)	17.4 (16.9–17.8)	50.7 (47.4–53.9)	17.1 (16.1–18.1)	47.9 (45.6–50.1)	19.3 (18.6–20.1)	44.2 (42.1–46.3)	15.8 (15.2–16.4)
14	49.3 (47.8–50.7)	19.0 (18.6–19.5)	53.7 (50.2–57.0)	18.9 (17.8–20.0)	50.0 (47.7–52.4)	20.9 (20.2–21.7)	45.6 (44.3–48.7)	17.5 (16.9–18.2)
16	51.7 (50.1–53.2)	20.7 (20.3–21.2)	57.3 (53.6–60.9)	20.9 (19.7–22.2)	52.9 (50.3–55.3)	22.6 (21.8–23.4)	48.4 (46.0–50.6)	19.2 (18.5–19.9)
18	53.3 (51.7–54.9)	22.3 (21.7–22.8)	58.2 (54.3–61.8)	22.9 (21.5–24.3)	54.4 (51.7–57.0)	24.4 (23.5–25.3)	50.0 (47.6–52.4)	20.4 (19.6–21.1)
20	55.1 (53.4–56.8)	23.6 (23.1–24.2)	59.5 (55.3–63.4)	24.2 (22.7–25.8)	56.5 (53.6–59.3)	25.8 (24.8–26.8)	52.2 (49.6–54.8)	21.7 (20.9–22.5)
22	56.1 (54.2–57.9)	25.0 (24.3–25.6)	61.2 (56.3–64.0)	26.4 (24.5–28.3)	57.7 (54.5–60.7)	26.8 (25.7–27.9)	53.2 (50.4–55.9)	23.1 (22.1–24.0)

For each of the nine categories of mental disorders, Table 3 shows HRs for patients compared to controls for the total sample and the three age groups 8–13, 14–17, and 18–32. For all mental groups and on each stratum, the HRs were larger when closer to inclusion while reduced to about two to three in the long term. For example, for any non‐ED mental disorder during the first 12 months the hazard ratios were HR = 17.4 (95% CI, 14.0–21.7) and HR = 5.04 (95% CI, 4.42–5.75) for patients aged 8–13 and 18–32 at inclusion, respectively, while at five or more years the two ratios had reduced to HR = 2.17 (95% CI, 1.78–2.65) and HR = 2.02 (95% CI, 1.77–2.31), respectively. During the first 2 years, a HR larger than nine was found for affective, obsessive–compulsive, and personality disorders in the age group 8–13. In the combined sample, large HRs ranging in 6–9 during the first years were found for affective, obsessive–compulsive, any non‐ED mental, autism spectrum, and personality disorders. There were no indications of non‐proportional hazards (*p* > .70) for any disorder. Differences across age groups pertained to any non‐ED mental, obsessive–compulsive, affective, and adjustment disorders at the first years after inclusion with higher HR for the age groups 8–13 and lower HR for the age group 18–32.

**TABLE 3:** Hazard ratios (HR) (95% CI) of other mental disorders during follow‐up for patients with anorexia nervosa compared to controls

	All	Age 8–13	Age 14–17	Age 18–32	Interaction
*N*	Patients *n* = 9,985, controls, *n* = 49,351	Patients *n* = 1724, controls, *n* = 8,519	Patients *n* = 3,809, controls, *n* = 18,848	Patients *n* = 4,452, controls, *n* = 21,984	
	HR^a^ (95% CI)	HR^a^ (95% CI)	HR^a^ (95% CI)	HR^a^ (95% CI)	
*Any non‐ED mental disorder*					
Months after inclusion: 0–12	7.47 (6.86–8.14)	17.4 (14.0–21.7)	8.29 (7.26–9.47)	5.04 (4.42–5.75)	*p* < .001
Years after inclusion: 1–4	3.25 (3.02–3.51)	3.59 (3.01–4.28)	3.05 (2.71–3.42)	3.43 (3.08–3.86)	^c^
Years after inclusion: 5+	2.05 (1.88–2.24)	2.17 (1.78–2.65)	2.04 (1.76–2.35)	2.02 (1.77–2.31)	^c^
*Substance use disorders*					
Years after inclusion: 0–1	2.45 (2.05–2.93)	2.36 (1.20–4.66)	2.42 (1.87–3.13)	2.54 (1.99–3.24)	^c^
Years after inclusion: 2–11	1.96 (1.75–2.19)	1.62 (1.26–2.08)	1.77 (1.50–2.10)	2.43 (2.05–2.89)	^c^
Years after inclusion: 12+	1.81 (1.38–2.37)	1.40 (0.66–2.97)	1.93 (1.15–3.22)	1.86 (1.30–2.67)	^c^
*Schizophrenia or psychosis*					
Years after inclusion: 0–3	4.60 (3.87–5.47)	6.43 (3.97–10.4)	5.17 (4.04–6.63)	3.96 (3.12–5.03)	^c^
Years after inclusion: 4+	2.74 (2.28–3.30)	3.38 (2.3–4.84)	2.38 (1.76–3.13)	2.82 (2.10–3.77)	^c^
*Affective disorders*					
Years after inclusion: 0–12	8.03 (6.86–9.41)	21.2 (12.5–35.8)	8.97 (7.15–11.25)	5.91 (4.73–7.37)	*p* < .001
Years after inclusion: 1–4	3.43 (3.07–3.83)	4.29 (3.19–5.76)	3.77 (3.20–4.43)	3.00 (2.55–3.52)	^c^
Years after inclusion: 5+	2.45 (2.22–2.69)	2.78 (2.24–3.45)	2.39 (2.04–2.79)	2.34 (2.03–2.71)	^c^
*Phobia or anxiety disorders*					
Years after inclusion: 0–5	3.07 (2.73–3.47)	3.68 (2.71–4.99)	2.84 (2.37–3.40)	3.15 (2.66–3.73)	^c^
Years after inclusion: 6+	2.25 (1.98–2.56)	2.59 (1.96–3.42)	1.91 (1.56–2.35)	2.43 (1.99–2.95)	^c^
*Obsessive–compulsive disorders*					
Years after inclusion: 0–2	8.02 (6.47–9.93)	16.0 (10.4–24.5)	7.30 (5.41–9.85)	5.01 (3.46–7.25)	*p* < .01
Years after inclusion: 3+	5.98 (4.95–7.22)	7.37 (4.96–11.0)	6.00 (4.49–8.02)	5.27 (3.91–7.11)	^c^
*Adjustment disorders+*					
Years after inclusion: 0–2	3.88 (3.50–4.31)	6.17 (4.87–7.80)	4.01 (3.45–4.66)	3.18 (2.71–3.74)	*p* < .001
Years after inclusion: 3+	2.28 (2.08–2.50)	2.87 (2.08–3.18)	2.24 (1.93–2.59)	2.23 (1.98–2.55)	^c^
*Personality disorders*					
Years after inclusion: 0–2	6.28 (5.52–7.15)	9.53 (5.81–15.7)	6.49 (5.29–7.96)	6.00 (5.08–7.07)	^c^
Years after inclusion: 3–12	3.83 (3.42–4.29)	3.27 (2.54–4.21)	3.76 (3.20–4.41)	4.15 (3.44–5.00)	^c^
Years after inclusion: 13+	2.93 (2.25–3.82)	3.75 (1.98–7.10)	3.28 (2.20–4.88)	2.40 (1.60–3.61)	^c^
*Autism spectrum disorders*					
Months after inclusion: 0–12	8.92 (6.06–13.0)	^b^	^b^	^b^	
Years after inclusion: 13	2.94 (2.25–3.86)	^b^	^b^	^b^	

^a^ Adjusted for period of inclusion, sex, any mental diagnoses in the child during the last 2 years prior to inclusion, family income at baseline (in tertiles), and previous mental disorders for parents at baseline (at least one diagnoses in at least one parent).

^b^ Too few ASD‐observations to fit proportional hazards model.

^c^ *p* > .05.

## 4 DISCUSSION

The present study used a large nationwide sample of patients with AN followed‐up with a median duration of 9.0 years. The findings contribute to the more limited findings based on previous observations made in follow‐up studies of rather small clinical samples with varying follow‐up periods, and mostly without any controls, that never considered analyses in a time‐to‐event setting.

In the present study, there was about a 25% and a 55% risk of receiving any non‐ED disorder for the patients during the first 2 years and two decades after inclusion, respectively. A HR of seven for any non‐ED was found for the first 12 months after inclusion, a ratio that reduced to two at five or more years after inclusion. During the first years after inclusion, large HRs ranging in 6–9 were found for affective, autism spectrum, personality, and obsessive–compulsive disorders with the latter displaying the highest continuous increased risk. The HR at 12 months after inclusion was highest for any non‐ED disorder and affective disorders in patients aged 8–13 at diagnosis.

The findings of this study reflect a pronounced overall diagnosed psychiatric comorbidity in AN across time. For patients, one out of three was assigned at least one mental disorder in the 4 years after receiving their first AN diagnosis, while it was only eight out of 100 for the controls. Cumulative incidences of eight major categories of mental disorders among patients showed notable increased risks over time for patients compared to controls. The immediate risks were always at double or more when compared to controls and for some disorders with much larger HR when close to inclusion, particularly for the age group 8–13. For example, for any non‐ED the HR was at seven during the first 12 months after inclusion and reduced to two at 5 years after inclusion. Close to inclusion, controls were at lower risk of any non‐ED at young age than at older age, while a subdued but reversed pattern was found for the patients, leading to particular high HR close to inclusion for the age group 8–13.

This marked burden imposed on individuals with AN by additional mental disorders at the time of, and soon after, the first AN diagnosis is reflected in Figure 2 by the shape of the curves for cumulative incidences. Among the matched controls, the cumulative incidences increased to about 10% after 20 years for affective and adjustment disorders and were lower for the remaining six disorders.

The steep increase in risk of subsequent mental disorders in all three age groups of patients with AN, and the high immediate risk when compared to controls, near the time of inclusion is in parallel to the often life‐threatening and highly complicated clinical picture of AN at first presentation. These observations about the temporal sequence provide information that goes beyond the findings of a heightened general risk of comorbid mental disorders as generated by previous studies (Demmler et al., 2020; Hudson et al., 2007; Keski‐Rahkonen & Mustelin, 2016; Marucci et al., 2018; Steinhausen, 2002; Tsai et al., 2018; Udo & Grilo, 2019; Ulfvebrand et al., 2015) and indicate that AN in most instances is a multifaceted disorder that requires complex interventions. On the other hand, these findings correspond to the recent conclusion from a Danish national population study that comorbidity within mental disorders is pervasive (Plana‐Ripoll et al., 2019).

In addition to these general patterns of cumulative incidences, age at diagnosis proved to have a relevant impact on the occurrence of comorbid disorders for patients with AN. It was particularly the youngest age group with a diagnosis of AN as early as age 8–13 that had the highest hazards when compared to controls for any non‐ED, obsessive–compulsive disorders, affective disorders, and adjustment disorders while the ratios were lower for those who were diagnosed only later at age 18–32. While it cannot be ruled out that different traditions and concepts of training in assessment as performed by child and adolescent psychiatrists versus adult psychiatrists may have contributed to this age effect, these findings point to the high vulnerability of the youngest group and also represent a more precise definition of the age at risk than described before in the literature on outcome of AN (Steinhausen, 2002). Obviously, the common distinction of age at onset in adolescence, that is, <18 years of age, versus adult age is not precise enough. However, it has to be admitted that the common reference to age at onset with its retrospective nature of definition may not have been used precisely enough in many of the older studies so that the term of age at diagnosis used in the present study is definitely more valid.

Furthermore, our study found that the hazard of being assigned a comorbid psychiatric diagnosis was declining in patients with AN compared to controls over a 20‐year period after inclusion. The HRs declined with length of follow‐up time, indicating, in line with the shape of the cumulative incidence curves, that the burden of comorbid disorders on patients with AN tends to decrease with longer duration of the main disorder but that the risk over 20 years is always at double or more. This finding represents an important extension of the observation that a large proportion of patients with AN suffered from comorbid mental disorders at single follow‐up examinations in the most comprehensive review of studies on the course of the disorder (Steinhausen, 2002).

In summary, the present findings may be regarded as coming from the most representative presentation of various comorbid disorders of AN in a single study. They extend the findings of a series of previous studies that focused predominantly on the prevalence rather than the incidence of single disorders in relatively small samples either at clinical intake or in uncontrolled follow‐ups (Carrot et al., 2017; Fouladi et al., 2015; Franko et al., 2018; Herpertz‐Dahlmann et al., 2018; Koch et al., 2015; Martinussen et al., 2017; Meier et al., 2015; Nazar et al., 2016; Root et al., 2010; von Lojewski et al., 2013; Wentz et al., 2009; Westwood & Tchanturia, 2017; Yilmaz et al., 2020) so that direct comparisons with the results of these studies are hampered.

It is noteworthy that the findings of the present study converge with the idea of genetic overlaps between AN and various comorbid psychiatric conditions. In fact, a recent genome‐wide association study (GWAS) showed significant correlations of AN with psychiatric disorders (Watson et al., 2019). Further strengths of the present study are the aforementioned characteristics of the design with large nationwide samples including patients and well‐matched controls and long median follow‐up. Free access to psychiatric institutions without individual costs as a part of the national health service and GPs referring patients with AN and other mental disorders mostly to psychiatry contribute to the representativeness of the samples. Furthermore, the findings were adjusted for various potential confounders including age, sex, area of residence, previous mental diagnoses of AN patients and controls, period of inclusion, family income at baseline, and previous mental disorders of parents.

Limitations include the structure of the NPR in general and the DPCRR in particular. These registries do not contain information at discharge or at the end of the treatment period. Thus, no precise information on the course of AN is available, and therefore questions regarding rates of recovery, improvement, chronicity or general outcome of AN cannot be answered by the present study. However, the focus of the present study was on comorbidity rather than on outcome of AN. Furthermore, because ICD classifies ED differently to the Diagnostic and Statistical Manual (DSM) and the information on the subtype of AN in the register can be ambiguous, the association of bulimic features in AN with other comorbid psychopathology cannot be tested. Other issues not addressed by the present study are the stability of the AN diagnosis across follow‐up time, the rates of diagnostic crossover within the spectrum of ED, and the impact of the clinical setting in which the first observed AN occurred.

Furthermore, the steep increase in the cumulative incidence functions of comorbid mental disorders in the patients with AN may indicate increased surveillance for other disorders at inclusion and shortly following the first AN diagnosis, but not later in time. However, concurrent comorbidity may also match the severity of the patient symptoms at this time during the course of AN as many of these patients have a protracted onset of treatment that may increase severity and chronicity of the disorder (Lock, La Via, & American Academy of Child and Adolescent Psychiatry Committee on Quality Issues, 2016). In the controls, there was also a steady increase in non‐ED mental disorders but on a much smaller level than in the patients. However, one might argue that the rather strict inclusion criterion in the controls of not having an ED diagnosis at any time of the follow‐up period might have resulted in a bias toward lower morbidity rates in this sample. Given the rather low incidences of ED in the general Danish population (Mohr‐Jensen, Vinkel Koch, Briciet Lauritsen, & Steinhausen, 2016), the number of controls with an ED diagnosis later in time than at the age of diagnosis of the corresponding patients with AN would have been rather small if a less strict criterion would have been employed.

While we studied the entire range of mental disorders, the absence of hyperkinetic disorders is noteworthy. The awareness of this comorbidity may still have been insufficient during our study period, particularly in adult patients (Svedlund, Norring, Ginsberg, & von Hausswolff‐Juhlin, 2017) as the frequency of hyperkinetic disorders were <5 in our data, meaning that the data could not be included in the analyses due to legislation on anonymization of data from the Danish registers.

Other concerns related to studies based on registries are less limiting. There was no independent verification of the accuracy of diagnoses entered in the DCPRR in the present study, although prior quality checks on the DCPRR suggest that diagnostic validity is high across various disorders (Kessing, 1998; Lauritsen et al., 2010; Loffler et al., 1994; Mohr‐Jensen et al., 2016). However, validity studies using structured diagnostic interviews with the patients are missing. It should be kept in mind that the AN group in the data set was determined by treatment seeking and referral to hospital‐based services. Given that not all patients with ED seek treatment, it can be assumed that a number of patients with AN may have contributed to a selection bias of unclear amount with an over‐representation of comorbid disorders in the patient sample. Furthermore, all patients were treated in the public health system. There is no obligation to register data on patients treated in the rather small sector of privately practising physicians. Finally, there was an unknown but likely small number of individuals that were lost during follow‐up due to emigration. Future studies may build from the present findings and explore and disentangle concurrent and sequential comorbidity, also focusing on the temporal stability and predictive validity of a comorbidity‐based approach to ED classification.

In conclusion, the findings of the present study document the excessive burden of comorbid mental disorders imposed on patients suffering from AN. Clinically, both assessment and interventions of comorbid mental disorders should be integrated into a highly complex plan that goes beyond the mere consideration of AN itself. This multi‐dimensionality of care is mandatory for preventing life‐damaging courses of the disease in the afflicted patients.

## CONFLICT OF INTEREST

In the past 3 years, HCS has worked as a speaker for Medice and has received book royalties from Cambridge University Press, Elsevier, Hogrefe, Klett, and Kohlhammer publishers. No other disclosures were reported.

## Supporting information

**Table S1** Other mental disorders for patients with anorexia nervosa and controls during follow‐up.

## Data Availability

Data were provided by Statistics Denmark in an anonymized fashion by request of the authors.
