# Supplementary material for: Comorbid mental disorders during long‐term course in a nationwide cohort of patients with anorexia nervosa
Source: Int J Eat Disord. 2021 Jun 18;54(9):1608–18. doi: 10.1002/eat.23570 (PMC8453938; doi:10.1002/eat.23570)
Supplement: Supplementary file 1 — Table S1 Other mental disorders for patients with anorexia nervosa and controls during follow‐up. [file EAT-54-1608-s001.docx]

## Table S1. Other mental disorders for patients with anorexia nervosa and controls during follow-up

|  | **All** | | **Age 8–13 at inclusion** | | **Age 14–17 at inclusion** | | **Age 18–32 at inclusion** | |
| --- | --- | --- | --- | --- | --- | --- | --- | --- |
|  | Patients, n=9985 | Controls, n=49351 | Patients,  n=1724 | Controls,  n=8519 | Patients,  n=3809 | Controls,  n=18848 | Patients,  n=4452 | Controls,  n=21984 |
| Any non-ED mental disorder, no. (%) | 5804 (58.1) | 7080 (14.3) | 995 (57.7) | 1139 (13.3) | 2200 (57.8) | 2942 (15.6) | 2609 (58.6) | 2996 (13.6) |
| Substance use disorders, no. (%) | 927 (9.3) | 1737 (3.5) | 106 (6.1) | 289 (3.4) | 333 (8.7) | 769 (4.1) | 488 (11.0) | 677 (3.1) |
| Schizophrenia or psychosis, no. (%) | 709 (7.1) | 653 (1.3) | 99 (5.7) | 100 (1.2) | 251 (6.6) | 265 (1.4) | 359 (8.1) | 287 (1.3) |
| Affective disorders, no. (%) | 2674 (26.8) | 2732 (5.5) | 370 (21.5) | 368 (4.3) | 1045 (27.4) | 1118 (5.9) | 1259 (28.3) | 1245 (5.7) |
| Phobia or anxiety disorders, no. (%) | 1257 (12.6) | 1612 (3.3) | 181 (10.5) | 243 (2.8) | 421 (11.1) | 678 (3.6) | 655 (14.7) | 696 (3.1) |
| Obsessive-compulsive disorders, no. (%) | 796 (8.0) | 379 (0.8) | 207 (12.0) | 71 (0.8) | 294 (7.7) | 159 (0.8) | 295 (6.6) | 149 (0.7) |
| Adjustment disorders, no. (%) | 1931 (19.3) | 2527 (5.1) | 345 (20.0) | 396 (4.6) | 784 (20.6) | 1063 (5.6) | 802 (18.0) | 1068 (4.9) |
| Personality disorders, no. (%) | 1863 (18.7) | 1453 (2.9) | 172 (10.0) | 195 (2.3) | 611 (16.0) | 609 (3.2) | 1080 (24.3) | 647 (3.0) |
| Autism spectrum disorders, no. (%) | 333 (3.3) | 210 (0.4) | 99 (5.7) | 59 (0.7) | 177 (4.6) | 112 (4.6) | 57 (1.3) | 37 (0.2) |

ED = eating disorder
